# Unraveling the Role of Epicardial Adipose Tissue in Coronary Artery Disease: Partners in Crime?

**DOI:** 10.3390/ijms21228866

**Published:** 2020-11-23

**Authors:** Glória Conceição, Diana Martins, Isabel M. Miranda, Adelino F. Leite-Moreira, Rui Vitorino, Inês Falcão-Pires

**Affiliations:** Cardiovascular R&D Centre (UnIC), Department of Surgery and Physiology, Faculty of Medicine, University of Porto, 4200-319 Porto, Portugal; glorialmeida6100@gmail.com (G.C.); dlfm94@gmail.com (D.M.); imiranda@med.up.pt (I.M.M.); amoreira@med.up.pt (A.F.L.-M.); rvitorino@ua.pt (R.V.)

**Keywords:** coronary artery disease, epicardial adipose tissue, inflammation, cytokines

## Abstract

The role of epicardial adipose tissue (EAT) in the pathophysiology of coronary artery disease (CAD) remains unclear. The present systematic review aimed at compiling dysregulated proteins/genes from different studies to dissect the potential role of EAT in CAD pathophysiology. Exhaustive literature research was performed using the keywords “epicardial adipose tissue and coronary artery disease”, to highlight a group of proteins that were consistently regulated among all studies. Reactome, a pathway analysis database, was used to clarify the function of the selected proteins and their intertwined association. SignalP/SecretomeP was used to clarify the endocrine function of the selected proteins. Overall, 1886 proteins/genes were identified from 44 eligible studies. The proteins were separated according to the control used in each study (EAT non-CAD or subcutaneous adipose tissue (SAT) CAD) and by their regulation (up- or downregulated). Using a Venn diagram, we selected the proteins that were upregulated and downregulated (identified as 27 and 19, respectively) in EAT CAD for both comparisons. The analysis of these proteins revealed the main pathways altered in the EAT and how they could communicate with the heart, potentially contributing to CAD development. In summary, in this study, the identified dysregulated proteins highlight the importance of inflammatory processes to modulate the local environment and the progression of CAD, by cellular and metabolic adaptations of epicardial fat that facilitate the formation and progression of atherogenesis of coronaries.

## 1. Introduction

The ever-growing health and socioeconomic burden related to obesity have gathered efforts aimed at revealing the complex association between adipose tissue and cardiovascular disease [1]. Interest in organ-specific adiposity is rapidly increasing as a substantial amount of scientific-based evidence suggests that adipose tissue anatomic specificity is crucial to the pathophysiology of cardiometabolic and endocrine diseases [2]. In this context, epicardial adipose tissue (EAT) has emerged as an exciting fat depot due to its location, peculiar metabolic properties, and clinical measurability [3]. Indeed, several studies have recognized EAT to be an independent predictor of coronary artery disease (CAD) [4,5]; however, the nature of this association remains to be clarified. Thus, this study aims to analyze the current literature focusing on the molecular signature of EAT derived from CAD patients. Our goal is to provide an overview of the potential impact of dysfunctional EAT on CAD pathophysiology.

### 1.1. Coronary Artery Disease

CAD is one of the most common forms of heart disease and a serious health problem worldwide. CAD can lead to myocardial ischemia, myocardial infarction, heart failure, and ultimately to death. CAD is a severe chronic disease, characterized by progressive atherosclerotic occlusion of the coronary arteries, resulting in a mismatch between myocardial oxygen demand and supply [6]. Atherosclerosis is described as a low-grade inflammatory state of the intima of medium-sized arteries that is accelerated by well-known risk factors such as hypertension, diabetes, obesity, and dyslipidemia [7]. In most observational studies, overweight/obesity has been associated with an increased prevalence of CAD, suggesting that it is the major risk factor associated with the pathophysiology and progression of the disease [8].

**Figure 1:**
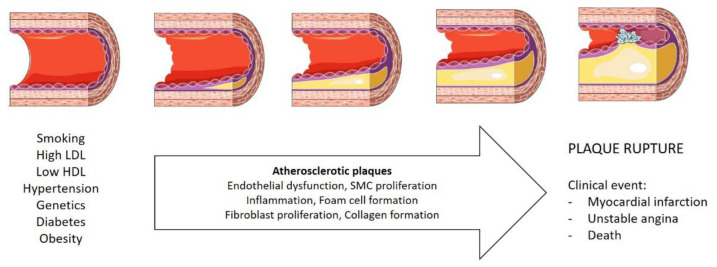
Coronary artery disease progression. LDL, low-density lipoproteins; HDL, high-density lipoproteins; SMC, smooth muscle cells. The figure was produced using Servier Medical Art.

Moreover, the risk of developing CAD is not the same for individuals with the same percentage of body fat, which is mainly caused by the different distributions of fat. Patients with visceral obesity develop CAD quickly, demonstrating that this fat pad can predict CAD onset [9]. Figure 1 illustrates an overview of atherosclerosis pathophysiology and progression in CAD. More information can be consulted elsewhere [10,11].

### 1.2. Obesity as a Risk Factor for Coronary Artery Disease

Obesity is a worldwide epidemic, representing a public health concern. Obesity is determined by a body mass index (BMI) above 30 kg/m^2^ [12]. However, is it widely known that BMI is a weak measurement of body fat, being influenced by muscle mass, body water content, and other factors. Furthermore, the relative contribution and burden of central, total, or subcutaneous adiposity to cardiovascular diseases needs further clarification. In lean subjects, subcutaneous adipose tissue (SAT) represents approximately 80% of the total adipose tissue mass, while visceral adipose tissue constitutes 15%. In obese patients, the percentage of visceral adipose tissue increases significantly, representing the most active fat subtype, which secretes adipocytokines that contribute to a systemic proinflammatory state and promote the development of cardiovascular atherosclerosis [13]. Moreover, visceral fat has the highest risk of metabolic dysregulation as a consequence of obesity, type II diabetes mellitus, or insulin resistance [14].

### 1.3. Epicardial Adipose Tissue

EAT is the fat depot that confers mechanical protection to the heart. It is directly connected to the myocardium, without any separating fascia, and shares the same circulation and blood supply [3]. EAT displays metabolic, thermogenic, and mechanical properties, with the higher rates of lipogenesis and fatty acid metabolism as compared to other fat subtypes. This enrichment and increased metabolism of free fatty acids (FFA) can be functionally important because the heart mostly depends on FFA oxidation as a source of energy [3]. Moreover, as an endocrine organ, EAT is the source of several bioactive adipocytokines that can either protect or adversely affect the myocardium and coronary arteries. Under normal physiological conditions, EAT can trigger cardioprotective actions through paracrine or vasocrine secretion of anti-inflammatory adipocytokines, such as adiponectin. However, upon adipocytes dysfunction, the balance of epicardial fat secretome is disrupted, the production and secretion of protective adipocytokines declines, while the release of proinflammatory adipocytokines through epicardial adipocytes increases [3].

### 1.4. Epicardial Adipose Tissue and Coronary Artery Disease (CAD)

Currently, several imaging techniques are used to effectively quantify epicardial fat, such as magnetic resonance imaging, transthoracic echocardiography, and cardiac computed tomography [15]. Several populational studies have extensively described the predictive and associative impact of the thickness/volume of epicardial fat on the development and progression of CAD [3,16]. An increasing number of studies has shown that EAT volume was consistently associated with visceral obesity and metabolic syndrome, and potentially represented a marker of CAD in asymptomatic high-risk patients [17,18]. Consequently, the interest in studying EAT volume as a predictor of CAD has increased, demonstrating that it can be a useful marker of CAD in asymptomatic patients with noncalcified plaques and zero calcium scores. Interestingly, EAT significantly correlates with CAD development, independently of the existence of cardiovascular risk factors or the volume of other fat depots [19]. Accordingly, EAT volume correlated with coronary calcification independently of global and visceral abdominal adiposities in a cohort of stable elderly patients [20], supporting the idea that EAT could be involved in all stages of CAD.

Some studies have favored the idea that EAT facilitated coronary atherosclerosis directly through an imbalance between cardioprotective and deleterious adipocytokines secreted. These studies strongly supported adipocytokines paracrine rather than systemic effects [14]. Additionally, EAT from CAD patients have shown more interaction and adherence between cells and cell-to-matrix, an increased inflammatory response through the infiltration of complement factors and platelets, as well as dysfunction of lipid metabolism and mitochondria [21].

## 2. Methods

### 2.1. Search Strategy

Records that were published up to December 2019 were retrieved from the PubMed database. The keywords “epicardial adipose tissue” were combined with “coronary artery disease” for the search. Two authors (G.C. and D.M.) independently screened records, compared the results, and discussed discrepancies to obtain consensus at each step based on the criteria of study selection.

### 2.2. Inclusion and Exclusion Criteria of Study Selection

Studies conducted in CAD patients that performed molecular studies in EAT were included. Case reports, conference/dissertation abstracts, echocardiographic and clinic studies, animal model studies, literature reviews, and in vitro experiments were excluded.

### 2.3. Data Extraction

Data from the reports were manually curated and organized to extract all genes and proteins that could be identified and whose variation had been assessed between the different conditions. Only studies that reported significant differences (*p* < 0.05) were included. We separated the studies based on the control used (SAT) from CAD patients and EAT from non-CAD patients). The genes/proteins identified were separated by their regulation (up- or downregulated) as compared with a selected control. This resulted in four different lists of proteins/genes, namely: (a) the proteins/genes upregulated in EAT CAD as compared with EAT non-CAD, (b) the proteins downregulated in EAT CAD as compared with EAT non-CAD, (c) the proteins upregulated in EAT CAD as compared with SAT CAD, and (d) the proteins downregulated in EAT CAD as compared with SAT CAD.

### 2.4. Bioinformatic Analysis

The identified genes and proteins were analyzed using the following bioinformatics tools: (1) PANTHER database (http://www.pantherdb.org) was used to perform gene ontology (GO) analyses, based on the biological process; (2) FunRich tool (http://www.funrich.org) was used to construct Venn diagrams, to perform an integrative analysis of the proteins/genes between the different groups; (3) SignalP and SecretomeP bioinformatics analysis were performed to search for putative secreted proteins [22] to elucidate the endocrine function of EAT in CAD, i.e., SignalP predicts classically secreted proteins based on signal peptide triggered protein secretion and SecretomeP predicts non-classical secreted proteins; and (4) Reactome, an open-source, peer-reviewed pathway analysis database [23], was used to clarify the relevance and to further explore the function of the proteins and their intertwined associations (http://reactome.org.). The set of selected genes from the Venn diagram and SignalP/SecretomeP analysis were placed into the “analysis” section of reactome. The program matches these proteins/genes to pathways and provides a pictogram of significant pathways (see Figure S1). The Reactome analysis program lists entities found in each pathway, along with a ratio of those genes found versus total molecules in the pathway, with a *p* value signifying “overrepresentation”, i.e., a larger number than would be expected if the set were random, with a Benjamin–Hochberg correction. Only the top 10 entities in the upregulated and the downregulated groups were detailed. The list also includes a false discovery rate (FDR) for each entity, indicating the expected proportion of rejected genes that were incorrect rejections.

## 3. Results

**Figure 2:**
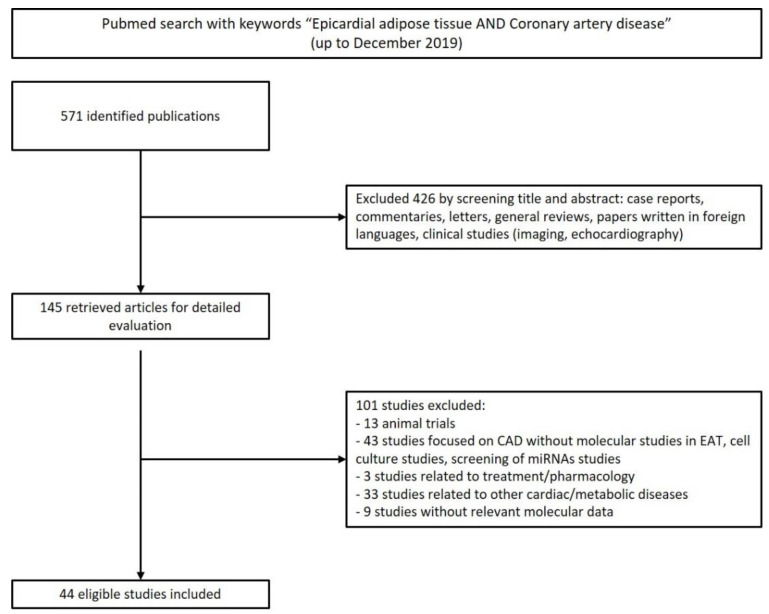
Search strategy flowchart. From the 571 abstracts collected in PubMed, using the keywords “epicardial adipose tissue and coronary artery disease”, 44 reports were used for the systematic review and 527 were excluded, according to the criteria above mentioned. CAD, coronary artery disease; EAT, epicardial adipose tissue.

Over 571 abstracts were retrieved and reviewed, taking into account the exclusion and inclusion criteria, achieving 44 valid reports for further analysis (Figure 2). In a total of 44 studies, the average of participants ranged from 45 to 74 years. Mostly, EAT samples were collected adjacent to the right coronary artery to perform molecular studies, such as polymerase chain reaction (PCR), Western blot, and other methods. The characteristics for each paper analyzed and a list of the proteins identified can be found in Table S1.

**Figure 3:**
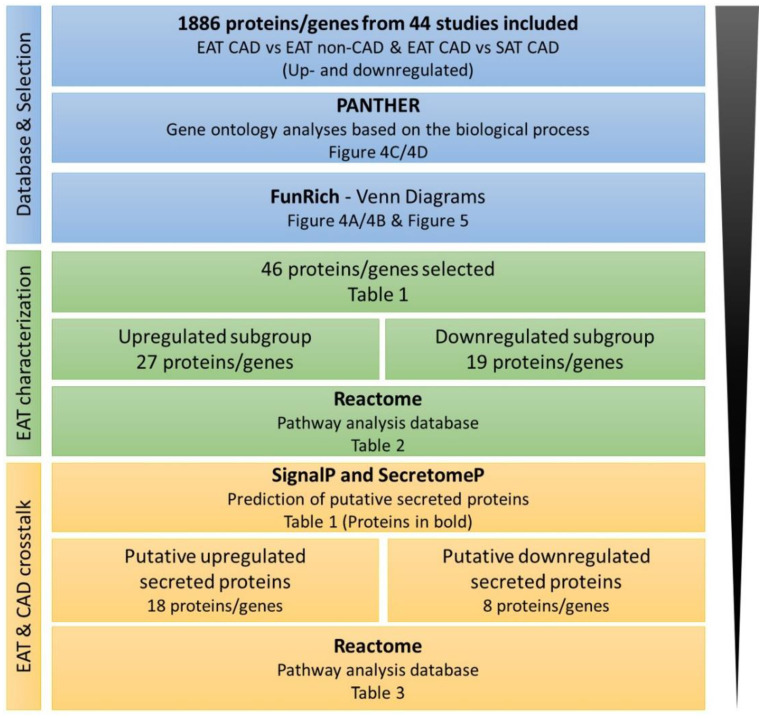
The workflow for proteins/genes analysis using bioinformatic tools. SAT, subcutaneous adipose tissue.

**Figure 4:**
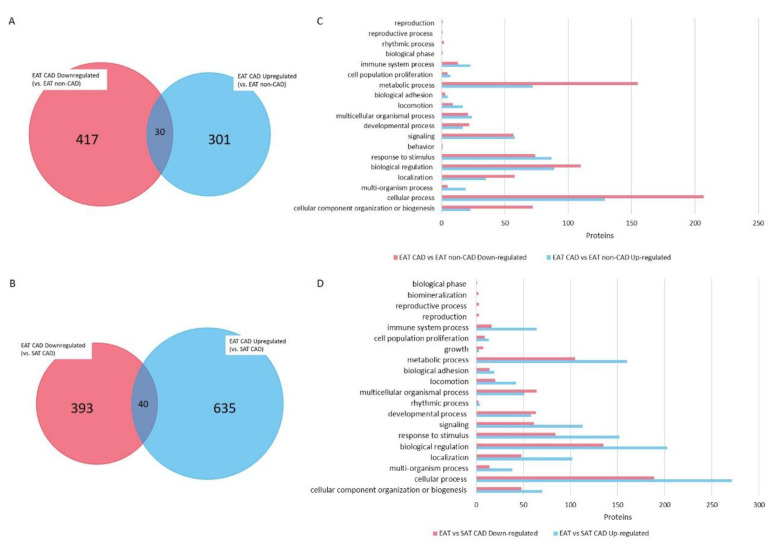
Venn diagram representing the distribution of identified proteins per control evidencing the overlapped and unique proteins. Proteins identified in EAT from studies using EAT non-coronary artery disease (non-CAD) as control (**A**) and corresponding altered biological processes (**C**); Proteins identified in EAT from studies using SAT CAD as control (**B**) and corresponding altered biological processes (**D**).

From these 44 eligible studies, 1886 proteins/genes were identified as dysregulated in EAT and subjected to bioinformatic analysis to filter the most relevant information, as summarized in Figure 3. From these, 1108 were identified from studies with SAT CAD as the control and 778 were recognized from studies EAT CAD versus EAT non-CAD (Table S2). Figure 4A,B illustrates the expression pattern of each one of these comparisons. From all proteins identified in EAT as compared with EAT from non-CAD patients, 301 proteins were described as upregulated and 417 proteins as downregulated. Contrarily, 30 proteins were inconsistently regulated. Relative to EAT proteins with SAT CAD as the control, 635 proteins were upregulated and 393 proteins were downregulated, and 40 proteins were inconsistently regulated.

For further understanding of the different proteins differentially identified, the proteins were queried in the PANTHER database v15.0 and annotated GO terms based on biological processes. The classification results are illustrated in Figure 4C,D. Independent of the group used as the control, the 3 main biological processes in EAT CAD with more proteins were metabolic process (GO:0008152, *p* = 6.10 × 10^−29^ vs. EAT non-CAD and *p* = 1.83 × 10^−12^ vs. SAT CAD), cellular process (GO:0009987, *p* = 2.93 × 10^−28^ vs. EAT non-CAD and *p* = 9.99 × 10^−16^ vs. SAT CAD), and biological regulation (GO:0065007, *p* = 4.12 × 10^−19^ vs. EAT non-CAD and *p* = 6.13 × 10^−29^ vs. SAT CAD). In addition, response to stimulus (GO:0050896, *p* = 1.55 × 10^−57^ vs. EAT non-CAD and *p* = 4.90 × 10^−37^ vs. SAT CAD), cellular component organization or biogenesis (GO:0071840, *p* = 4.56 × 10^−8^ vs. EAT non-CAD and *p* = 1.41 × 10^−6^ vs. SAT CAD), localization (GO:0051179, *p* = 1.79 × 10^−21^ vs. EAT non-CAD and *p* = 6.71 × 10^−21^ vs. SAT CAD), signaling (GO:0023052, *p* = 4.48 × 10^−28^ vs. EAT non-CAD and *p* = 3.43 × 10^−26^ vs. SAT CAD), developmental process (GO:0032502, *p* = 6.85 × 10^−6^ vs. EAT non-CAD and *p* = 4.18 × 10^−36^ vs. SAT CAD), multicellular organismal process (GO:0032501, *p* = 2.3 × 10^−16^ vs. EAT non-CAD and *p* = 1.46 × 10^−38^ vs. SAT CAD), and immune system process (GO:0002376, *p* = 3.73 × 10^−29^ vs. EAT non-CAD and *p* = 1.25 × 10^−27^. SAT CAD) were also consistently highlighted.

**Figure 5:**
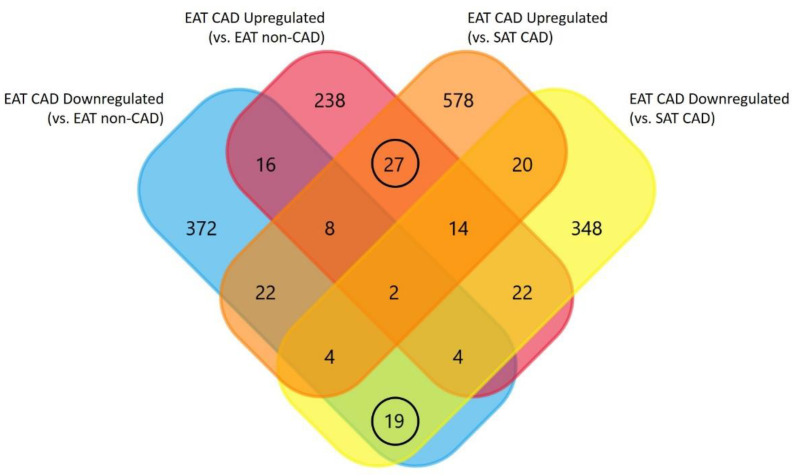
Venn diagram representing the distribution of proteins per control evidencing the overlapped and unique proteins in EAT, using the protein up- and downregulated in EAT CAD as compared with EAT non-CAD and SAT CAD.

A Venn diagram representing the differences in protein expression among groups was designed to highlight the most significant proteins underlying the interaction between EAT and CAD (Figure 5 and Table S3). This diagram identifies which proteins were simultaneously upregulated (27 in Figure 5, upregulated subgroup) and simultaneously downregulated (19 in Figure 5, downregulated subgroup) in EAT CAD for both comparisons (Table 1). On the one hand, from the upregulated proteins, one should highlight tumor necrosis factor, C-C motif chemokine 2, C-C motif chemokine 5, and interleukin (IL)-18. On the other hand, from the downregulated proteins, galectin-3, gelsolin, cathepsin K, and macrophage scavenger receptor types I and II should be emphasized.

## 4. Bioinformatics Analysis Provides a Protein Network Overview from CAD Epicardial Adipose Tissue

### 4.1. Functional Protein Categorization and Integrative Analysis with REACTOME

To further understand the biological implications of EAT in CAD condition, we performed network enrichment analysis using Reactome. The data revealed that upregulated proteins were integrated into 59 pathways and downregulated proteins were integrated into 47 pathways, with a *p* value restricted to ≤ 0.05.

The most representative pathways for the upregulated subgroup of proteins were interleukin-10 signaling (*p* = 8.66 × 10^−8^), signaling by interleukins (*p* = 8.91 × 10^−7^), immune system (*p* = 9.07 × 10^−7^), chemokine receptors bind chemokines (*p =* 1.15 × 10^−5^), and innate immune system (*p* = 6.06 × 10^−5^, Table 2). The most representative pathways for the downregulated subgroup of proteins were depolymerization of the nuclear (*p* = 4.05 × 10^−4^), scavenging by class A receptors (*p* = 5.7 × 10^−4^), initiation of nuclear envelope reformation (*p* = 6.3 × 10^−4^), apoptotic cleavage of cellular proteins (*p* = 2.23 × 10^−3^), and apoptotic execution phase (*p* = 4.11 × 10^−3^, Table 2).

### 4.2. Predictions of Putative Secreted Proteins

In order to add new insights regarding EAT and CAD crosstalk, SignalP and SecretomeP bioinformatics analysis of the upregulated and downregulated subgroups of proteins retrieved which proteins were putatively secreted (Table S5). These proteins are highlighted in bold in Table 1 and were reanalyzed with Reactome, showing similar results to those presented in Table 2. Briefly, upregulated secreted proteins were integrated into 47 pathways and downregulated secreted proteins were integrated into 39 pathways, with a *p* value restricted to ≤ 0.05. The most prevalent pathways associated with upregulated secreted proteins were interleukin-10 signaling (*p =* 8.91 × 10^−7^), signaling by interleukins (*p =* 5.33 × 10^−6^), immune system (*p =* 1.14 × 10^−5^), interleukin-4 and interleukin-13 signaling (*p =* 3.08 × 10^−5^), and interleukin-18 signaling (*p =* 1.05 × 10^−4^, Table 3). In contrast, the most prevalent pathways associated with downregulated secreted proteins were binding and uptake of ligands by scavenger receptors (*p* = 7.75 × 10^−5^), RUNX2 regulates genes involved in differentiation of myeloid cells (*p* = 8.6 × 10^−4^), neutrophil degranulation (*p* = 3.01 × 10^−4^), degradation of the extracellular matrix (ECM) (*p* = 3.41 × 10^−3^), and RUNX1 regulates transcription of genes involved in differentiation of myeloid cells (*p* = 3.5 × 10^−3^).

## 5. Discussion

This study was the first to compile dysregulated proteins/genes from different studies to scrutinize the potential role of EAT in CAD. Despite the significant amount of research focusing on the impact of EAT on CAD, controversial results still preclude a clear vision of this interaction. This partially results from the fact that some studies have different control groups, while other studies describe different proteins, even if related to the same biological processes. Lastly, some proteins are inconsistently regulated, i.e., being upregulated or downregulated, depending on the study (Figure 5). This inconsistency proves that the same protein can behave differently depending on other comorbidities that cannot be excluded from human studies. With this systematic review, we were able to highlight a group of proteins that were consistently regulated among all studies, accounting for the control used in each study (EAT CAD vs. EAT non-CAD or EAT CAD vs. SAT CAD). The analysis of these proteins revealed the main pathways altered in the EAT and how they could communicate with the heart, potentially contributing to CAD development.

### 5.1. The Contribution of Epicardial Adipose Tissue to the Proinflammatory Profile of CAD

The bioinformatic analysis, presented in this study, revealed innate and adaptive immunity activation as the most relevant signaling pathways in EAT from CAD patients. Accordingly, it is currently accepted that the adipose tissue represents an important and highly active part of the immune system [24]. Adipose tissue is composed of two distinct entities, i.e., adipocytes and the stromal-vascular fraction formed by ECM with dispersed fibroblasts, preadipocytes, endothelial and immune cells [25]. Adipose tissue-resident immune cells include almost the full spectrum of immune cell types, namely macrophages, B cells, T cells, neutrophils, eosinophils, and mast cells [24]. Cytokines and chemokines are released from a wide range of immune cells, as confirmed by our analysis (Table 1 and Table 2). These factors are indispensable for the communication between immune and non-immune cells and for the coordination of inflammatory responses, as well as the crosstalk between innate and adaptive immune system [26]. The chemokines overexpression, such as CCL2, CCL5, CCL13 and CCL18, triggers the recruitment of immune cells and the macrophages migration (Table 1). Macrophages plasticity endows their diverse activities in response to various environmental stimuli [27]. The M1 macrophages stimulate the conversion of unpolarized macrophages to M1 through the release of various proinflammatory and proatherogenic cytokines (IL-6 and TNF-α) and chemokines. For example, TNF-α initiates and amplifies inflammatory cascades through immune cells recruitment, chemokine regulation, and cytokine release, as well as the migration and mitogenesis of vascular smooth muscle and endothelial cells. Indeed, increased levels of TNF-α have been reported in EAT from CAD patients [28] (Table 1). Inversely, M2 macrophages can be activated by cytokines IL-4/IL-13, secreted from various cells in adipose tissue and are crucial to the promotion of preadipocyte survival, wound healing, and control of inflammation via anti-inflammatory cytokines production, such as IL-10, as evident in Table 2. Several studies have reported the influx of macrophages into the epicardial fat and have correlated the ratio of M1/M2 macrophages with the severity of CAD [29]. Indeed, excessive inflammation in CAD results from an imbalance between these two pathways, favoring M1 polarization and proinflammatory environment. Furthermore, other inflammatory pathways have been observed, such as IL-18 signaling, capable of increasing the atherosclerotic lesion size by T-lymphocytes attraction. Activated immune cells secrete several cytokines that influence adipocyte function and its paracrine secretion of cytokines (adipocytokines). Simultaneously, adipocytes produce inflammatory adipocytokines and ECM proteins, supporting infiltration and activation of immune cells. This vicious cycle creates an optimal microenvironment for low-grade inflammation, which underlies adipose tissue dysfunction.

Many studies have reported that CAD progression was closely associated with a higher volume of EAT, independently of obesity. Inflammation promotes adipose tissue expansion either through hypertrophy of existing adipocytes or differentiation of new adipocytes from adipogenic precursor cells (hyperplasia or adipogenesis) [30]. For instance, Sadler et al. showed that low-dose LPS led to adipocyte hyperplasia at the site of administration, associated with a net loss of adipose tissue collagen (Table 2), evidencing that the ability to effectively degrade ECM was essential for adipose tissue expansion [31,32]. In parallel to ECM remodeling, proinflammatory stimuli also promote angiogenesis in adipose tissue. These processes are both essential for adipogenesis in vivo [32] and explain the increased fat volume described in several studies [33]. Moreover, adipocytes hypertrophy is also associated with higher expression of proinflammatory adipocytokines, further perpetuating the vicious cycle of inflammation and fat expansion.

### 5.2. The Contribution of Epicardial Adipose Tissue to the Atherosclerotic Plaque Formation in CAD

Inflammation is also considered to be a central driver of atherogenesis and of the development of vulnerable atherosclerotic plaques [34,35]. Indeed, the proatherogenic effects of adipose tissue immune cells are carried out in the endocrine and paracrine manner by increased levels of the putative secreted proinflammatory proteins, as described above. Interestingly, we found IL-4, IL-10, and IL-13 signaling to be increased. We trust that the upregulation of these anti-inflammatory pathways might represent a compensatory mechanism to promote M2 polarization and to inhibit many cellular processes underlying plaque progression, rupture, or thrombosis, which include nuclear factor-κB activation, metalloproteinase proteinase production, tissue factor expression, and cell death. Further reinforcing this idea, other studies have shown that these factors were essential to keep the balance of the inflammatory response, to promote tissue repair, and to ensure plaque stability [36,37]. Interestingly, our analysis revealed reduced expression of cathepsin K and galectin-3 proteins, which have both been linked to the progression of atherosclerotic plaque. While cathepsin K has been associated with progression of unstable plaques and closely associated with CAD [38], galectin-3 has been shown to contribute to macrophage differentiation, foam cell formation, endothelial dysfunction, and vascular smooth muscle cells proliferation and migration [39] in the atheroma and its inhibition might reduce plaque progression [40]. Moreover, galectin-3 deficiency, in a mice model of atherosclerosis (ApoE^−/−^), decreased plaque size, its necrotic core, and collagen content [40], which was consistent with our findings (Table 1). The presence of collagen in the fibrous atherosclerotic plaque cape is essential to maintain its stability, avoiding plaque rupture [41]. In addition, we found the macrophage scavenger receptor class A and the transcript factor RUNX2 to be downregulated in EAT. Macrophage scavenger receptor class A is known to be involved in foam cell development, mediating the influx of lipids into the macrophages [42], and therefore knockout mice for this gene have shown lower proinflammatory responses, macrophage apoptosis, and cellular necrosis with better stabilization of atherosclerotic plaques [43].

Similarly, RUNX2 is activated by atherogenic factors such as lipid-derived products from oxidation present in valve lesions that can promote calcification and is increased in advanced calcified lesions, supporting their implication in active osteogenesis and mineralization of human atherosclerotic coronary arteries [44]. Nevertheless, RUNX2 downregulation seems to be crucial to keep EAT expansion in response to inflammation, once RUNX2 upregulation restrains adipogenesis [45]. The proteome of epicardial fat likely acts as a local trigger for coronary plaque growth, calcification and stability in different stages of CAD. Accordingly, an unbalance between calcification promoters and inhibitors in epicardial fat has been reported in advanced stages of CAD [20].

### 5.3. The Epicardial Adipose Tissue and Atheroma Communication

**Figure 6:**
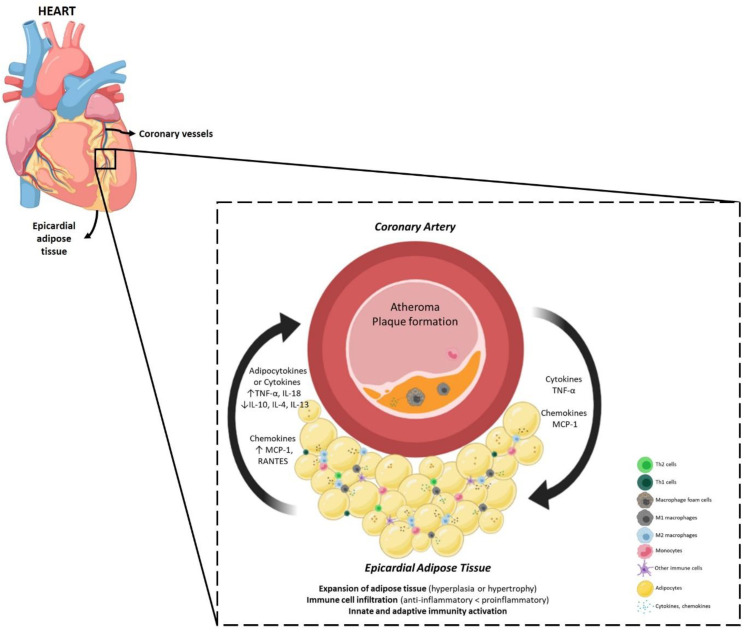
Interactions between EAT and atherosclerotic coronary arteries. In response to inflammatory cytokines released by atheroma, EAT can modulate the differentiation and function of adipocytes. EAT, located in close proximity with heart, can secrete adipocytokines which diffuse directly to the coronary arteries, promoting coronary inflammation. The figure was produced using BioRender and Servier Medical Art.

Notably, the cellular composition of adipose tissue is highly plastic and can be regulated by environmental acute and chronic stimuli [24]. This suggests that the signals arising from the cardiovascular system can modulate the differentiation and function of adipocytes, and consequently adipose tissue quality. This concept supports a bidirectional crosstalk between epicardial fat and the coronary arteries, as represented in Figure 6. Antonopoulos et al., described an increased expression of adiponectin in perivascular adipose tissue in response to the activation of NADPH oxidase in the human arterial wall using an ex vivo model of human internal mammary arteries and perivascular adipose tissue co-cultures [46]. Moreover, inflammation and alteration in adipokines expression were reported in perivascular adipose tissue of rats and pigs as a consequence of balloon-induced vascular injuries and drug-eluting stent-induced coronary vasoconstriction [47,48]. The inflammation present in atherosclerotic coronary arteries may contribute to attracting immune cells to EAT, as observed by the increased number of chemokines upregulated (Table 1 and Table 2). By doing so, the atheroma may affect the EAT composition and signaling.

### 5.4. Influence of Epicardial Adipose Tissue Proteome on Cardiac Function and Structure

Beyond the influence of EAT on CAD, EAT is also directly connected to the myocardium. Inflammatory mediators have significant repercussions in all myocardial cell types, namely in cardiomyocytes, fibroblasts, and endothelial cells [49,50]. The deleterious effects of the proinflammatory cytokines include myocardial cell death, blunted β-adrenergic signaling, fetal gene reactivation, endothelial dysfunction, and collagen deposition [49], which are major determinants in the pathogenesis of cardiac disorders. There is increasing evidence that proinflammatory adipocytokines trigger cardiomyocyte hypertrophy and apoptosis, fibroblast differentiation into active myofibroblasts, and adhesion of immune cells to the endothelium and trans-endothelial migration [49,51,52]. Moreover, experimental studies have shown that inflammation was responsible for left ventricular (LV) diastolic and endothelial dysfunction and progression towards heart failure with preserved ejection fraction (HFpEF) [53,54]. Cytokines such as TNF-α (Table 1) promote a direct negative inotropic effect, through downregulation of Ca^2+^-regulating genes including sarcoplasmic reticulum Ca^2+^ ATPase and Ca^2+^-release channel [55] and stimulate myofibroblasts activation [56,57]. Transgenic mice with cardiac-specific overexpression of TNF-α developed dilated cardiomyopathy with ventricular hypertrophy, ventricular dilatation, interstitial infiltrates, interstitial fibrosis, and reduced ejection fraction [58]. In contrast, IL-10 signaling suppressed the inflammatory response and contributed to improved LV function and remodeling (Table 2). Treatment approaches with IL-10 seem to be beneficial for preventing hypertrophy, reducing fibrosis, and preserving cardiac function, through the maintenance of cytokine homeostasis [59]. IL-4 and IL-13 signaling also modulate physiological processes, such as tissue repair, ECM remodeling, and metabolism homeostasis [60,61]. Additionally, we have observed a downregulation of RUNX1, that has been identified as a key regulator of adverse cardiac remodeling following myocardial infarction. RUNX1-deficient mice or RUNX1 knockout prevented adverse cardiac remodeling, restrained myocardial scar formation, and assured normal calcium homeostasis in cardiomyocytes [62]. Considering all the identified pathways, we believe that EAT fights to maintain cardiovascular homeostasis, orchestrating the inhibition and promotion of certain pathways.

### 5.5. Therapeutic Strategies against Inflammation-Related Coronary Artery Disease

Current pharmacological strategies for CAD patients include reducing angina symptoms, exercise-induced ischemia, and preventing cardiovascular events [63]. Taking into account the analysis presented in our study regarding the major impact of inflammation in CAD, therapeutic strategies should target the immune system with anti-inflammatory approaches [52]. Recently, the randomized clinical trial CANTOS assessed the effect of canakinumab, a human monoclonal antibody against IL-1β with anti-inflammatory properties. Canakinumab reduced significantly high-sensitivity C-reactive protein level from baseline in a dose-dependent fashion for three months, which persisted even after the treatment ended [64]. Moreover, the anti-inflammatory therapy targeting the IL-1β reduced the recurrence of cardiovascular events in well-treated CAD patients independent of any lowering effects on low-density lipoproteins (LDL) cholesterol levels [64].

## 6. Conclusions

The communication between EAT and CAD remains unclear. The dysregulated proteins, identified herein, highlight the importance of inflammatory processes for modulating the local environment and the progression of CAD. Inflammation triggers cellular and metabolic adaptations of epicardial fat that facilitate the formation and progression of atherogenesis of coronaries. Although several authors have supported that EAT thickness was a predictor for CAD, we also trust that the quality of epicardial fat should be assessed. For instance, to evaluate EAT-resident immune cells would ease the comprehension of the dynamic signaling at different time points of CAD progression.

In conclusion, future studies involving immune interventions should envisage clarifying the influence of anti-inflammatory drugs in EAT and how to modulate the paracrine and endocrine communication between epicardial fat and coronary arteries during CAD.

## Figures and Tables

**Table 1:** List of upregulated and downregulated proteins selected to characterize EAT. The putative secreted proteins are identified in bold.

Upregulated Subgroup (27 Proteins, 18 Secreted Proteins)	Downregulated Subgroup (19 Proteins, 8 Secreted Proteins)
**Tumor necrosis factor** Mitogen-activated protein kinase kinase kinase 8 Scavenger receptor cysteine-rich type 1 protein M130	Tyrosine-protein kinase ABL2 **5-aminolevulinate synthase**
**C-C motif chemokine 2** C-C chemokine receptor type 2	**erythroid-specific, mitochondrial** Amphiphysin
**Arachidonate 5-lipoxygenase-activating protein** **C-C motif chemokine 5**	**Aminopeptidase N**
Nitric oxide synthase, endothelial	**Cathepsin K**
Neutrophil cytosol factor 2 Histone-lysine N-methyltransferase PRDM16	Dynamin-1
**C-C motif chemokine 18**	**Gelsolin**
**Cathepsin E** **Leucine-rich repeat transmembrane protein FLRT3** **Interleukin-7 receptor subunit alpha**	**Macrophage scavenger receptor types I and II** Ubiquitin carboxyl-terminal hydrolase isozyme L1
**C-C motif chemokine 13** **T-cell surface glycoprotein CD3 zeta chain**	Butyrophilin-like protein 9 Zinc finger and BTB domain-containing protein 16
**HLA class I histocompatibility antigen protein P5** **Toll-like receptor 2**	**Galectin-3**
**Interleukin-18**	**Secreted frizzled-related protein 2**
E3 SUMO-protein ligase EGR2	Hormone-sensitive lipase
Early activation antigen CD69 L-selectin	Prelamin-A/C
**Alpha-1-antichymotrypsin**	Actin, gamma-enteric smooth muscle
**Complement C3**	**Collagen alpha-1(I) chain**
**Coronin-1A** **Intercellular adhesion molecule 3**	Acyl-CoA-binding protein
**Integrin beta-2**	Fructose-bisphosphate aldolase C

**Table 2:** The 10 most relevant pathways sorted by *p* value using the upregulated and downregulated subgroups of proteins. Entities found in each pathway are described in Table S4.

Pathway Name	Entities Found	Entities Total	Entities Ratio	Entities *p* Value	Entities FDR
Upregulated Proteins					
Interleukin-10 signaling	5	45	0.004	8.66 × 10^−8^	1.69 × 10^−5^
Signaling by Interleukins	9	456	0.04	8.91 × 10^−7^	5.9 × 10^−5^
Immune System	18	2398	0.21	9.07 × 10^−7^	5.9 × 10^−5^
Chemokine receptors bind chemokines	4	57	0.005	1.15 × 10^−5^	5.54 × 10^−4^
Innate Immune System	11	1187	0.104	6.06 × 10^−5^	2.36 × 10^−3^
Peptide ligand-binding receptors	5	198	0.017	1.1 × 10^−4^	3.53 × 10^−3^
Interleukin-4 and Interleukin-13 signaling	4	111	0.01	1.52 × 10^−4^	4.09 × 10^−3^
Interleukin-18 signaling	2	9	0.001	2.32 × 10^−4^	5.56 × 10^−3^
Adaptive Immune System	9	944	0.083	2.9 × 10^−4^	6.09 × 10^−3^
Cytokine Signaling in Immune system	9	981	0.086	3.86 × 10^−4^	7.34 × 10^−3^
Downregulated Proteins					
Depolymerisation of the Nuclear	2	16	0.001	4.05 × 10^−4^	2.65 × 10^−2^
Scavenging by Class A Receptors	2	19	0.002	5.7 × 10^−4^	2.65 × 10^−2^
Initiation of Nuclear Envelope (NE) Reformation	2	20	0.002	6.3 × 10^−4^	2.65 × 10^−2^
Apoptotic cleavage of cellular proteins	2	38	0.003	2.23 × 10^−3^	6.91 × 10^−2^
Apoptotic execution phase	2	52	0.005	4.11 × 10^−3^	8.17 × 10^−2^
Nuclear Envelope Breakdown	2	58	0.005	5.08 × 10^−3^	8.17 × 10^−2^
Breakdown of the nuclear lamina	1	3	0	5.5 × 10^−3^	8.17 × 10^−2^
Collagen degration	2	64	0.006	6.14 × 10^-3^	8.17 × 10^−2^
Nuclear Envelope (NE) Reassembly	2	78	0.007	8.99 × 10^−3^	8.17 × 10^−2^
RUNX2 regulates genes involved in differentiation of myeloid cells	1	5	0	9.15 × 10^−3^	8.17 × 10^−2^

Entities reflect proteins, small molecules and genes regarding the pathway. Entities *p* value indicates that the proteins within this pathway represent more than would be expected if the set were random, corrected for multiple testing (Benjamini–Hochberg) that arises from evaluation of the submitted list of identifiers against every pathway. FDR indicates false discovery rate corrected for the probability of over-representation.

**Table 3:** The 10 most relevant pathways sorted by *p* value using upregulated and downregulated proteins predicted to be secreted. Entities found in each pathway are described in Table S6.

Pathway Name	Entities Found	Entities Total	Entities Ratio	Entities *p* Value	Entities FDR
Upregulated Proteins					
Interleukin-10 signaling	4	45	0.004	8.91 × 10^−7^	1.08 × 10^−4^
Signaling by Interleukins	7	456	0.04	5.33 × 10^−6^	3.2 × 10^−4^
Immune System	13	2398	0.21	1.14 × 10^−5^	4.58 × 10^−4^
Interleukin-4 and Interleukin-13 signaling	4	111	0.01	3.08 × 10^−5^	9.23 × 10^−4^
Interleukin-18 signaling	2	9	0.001	1.05 × 10^−4^	2.27 × 10^−3^
Chemokine receptors bind chemokines	3	57	0.005	1.13 × 10^−4^	2.27 × 10^−3^
Peptide ligand-binding receptors	4	198	0.017	2.84 × 10^−4^	4.83 × 10^−3^
Cytokine Signaling in Immune system	7	981	0.086	6.86 × 10^−4^	9.59 × 10^−3^
Purinergic signaling in Leishmaniasis infection	2	25	0.002	8.00 × 10^−4^	9.59 × 10^−3^
Cell recruitment (proinflammatory response)	2	25	0.002	8.00 × 10^−4^	9.59 × 10^−3^
Downregulated Proteins					
Binding and Uptake of Ligands by Scavenger Receptors	2	19	0.002	7.7 × 10^−5^	4.85 × 10^−3^
RUNX2 regulates genes involved in differentiation of myeloid cells	2	64	0.006	8.6 × 10^−4^	2.58 × 10^−2^
Neutrophil degranulation	2	121	0.011	3.01 × 10^−4^	2.58 × 10^−2^
Degradation of the extracellular matrix	2	129	0.011	3.41 × 10^−3^	2.58 × 10^−2^
RUNX1 regulates transcription of genes involved in differentiation of myeloid cells	1	5	0	3.5 × 10^−3^	2.58 × 10^−2^
RUNX1 regulates transcription of genes involved in differentiation of keratinocytes	3	480	0.042	3.55 × 10^−3^	2.58 × 10^−2^
Innate Immune System	2	140	0.012	4.01 × 10^−3^	2.58 × 10^−2^
GP1b-IX-V activation signalling	1	8	0.001	5.59 × 10^−3^	2.58 × 10^−2^
Caspase-mediated cleavage of cytoskeletal proteins	1	8	0.001	5.59 × 10^−3^	2.58 × 10^−2^
Transcriptional regulation by RUNX1	4	1187	0.104	5.79 × 10^−3^	2.58 × 10^−2^

Entities reflect proteins, small molecules and genes regarding the pathway. Entities *p* value indicates that the proteins within this pathway represent more than would be expected if the set were random, corrected for multiple testing (Benjamini–Hochberg) that arises from evaluation of the submitted list of identifiers against every pathway. FDR indicates false discovery rate corrected for the probability of over-representation.

## Supplementary Materials

The following are available online at https://www.mdpi.com/1422-0067/21/22/8866/s1, Figure S1: Representative sketch results of enrichment analysis with Reactome, Table S1: Eligible studied included and the proteins identified, Table S2: List of proteins identified in EAT from studies using EAT non-CAD as control or SAT CAD as control, Table S3: Distribution of proteins by Venn diagram, Table S4: Entities found in the 10 most relevant pathways sorted by p value using upregulated and downregulated subgroups of proteins, Table S5: Signal P/SecretomeP report, Table S6: Entities found in the 10 most relevant pathways sorted by p value using the upregulated and downregulated subgroups of potentially secreted proteins.
