# Supplementary material for: Unraveling the Role of Epicardial Adipose Tissue in Coronary Artery Disease: Partners in Crime?
Source: Int J Mol Sci. 2020 Nov 23;21(22):8866. doi: 10.3390/ijms21228866 (PMC7700147; doi:10.3390/ijms21228866)
Supplement: Supplementary file 1 [file ijms-21-08866-s001.zip › Supplementary Figure.docx]

Supplementary Figure 1


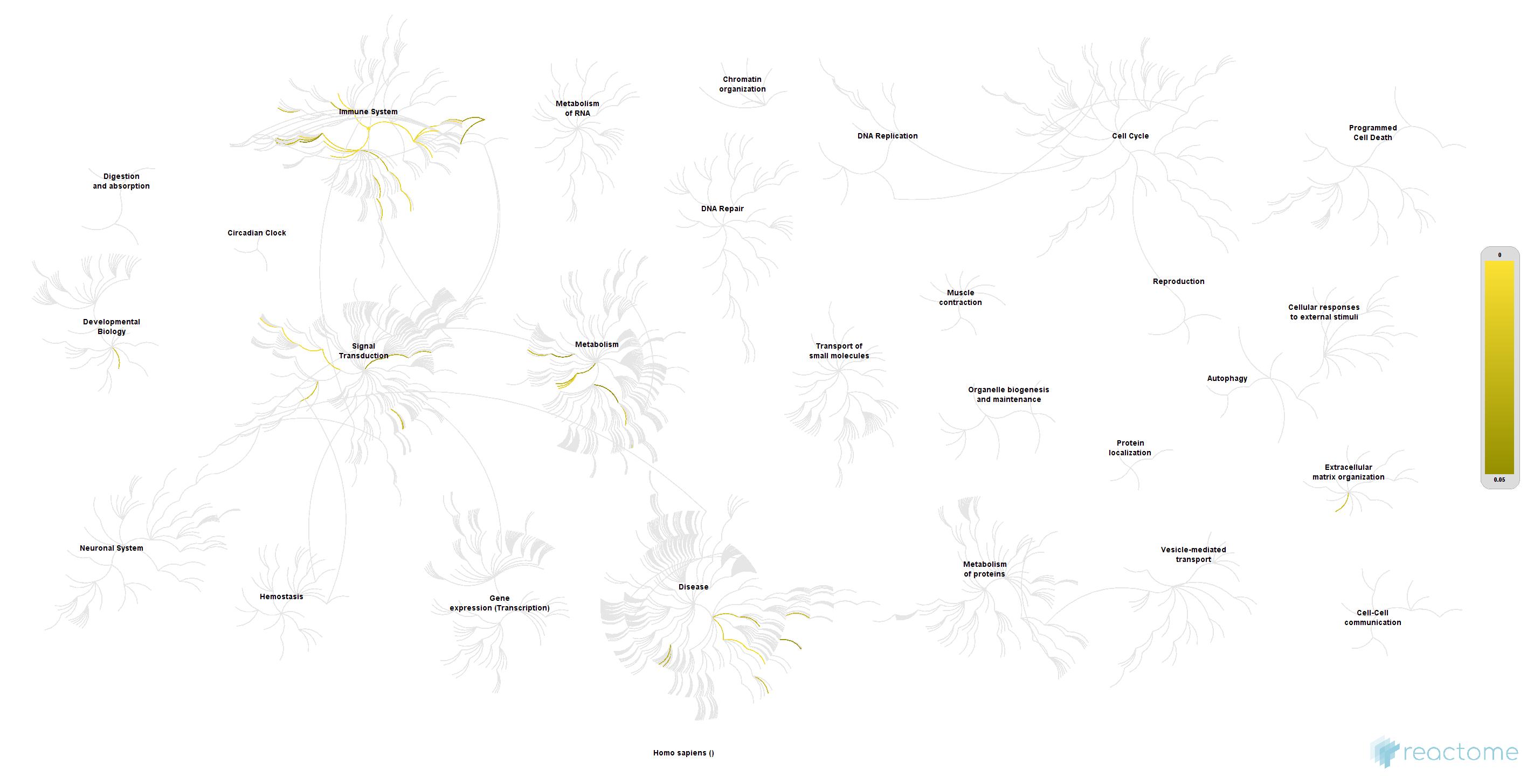


***Figure S1 -*** Representative sketch results of enrichment analysis with Reactome.
